# Kappa statistic considerations in evaluating inter-rater reliability between two raters: which, when and context matters

**DOI:** 10.1186/s12885-023-11325-z

**Published:** 2023-08-25

**Authors:** Ming Li, Qian Gao, Tianfei Yu

**Affiliations:** 1 Department of Computer Science and Technology, College of Computer and Control Engineering, Qiqihar University, Qiqihar, 161006 China; 2 Department of Biotechnology, College of Life Science and Agriculture Forestry, Qiqihar University, Qiqihar, 161006 China

**Keywords:** RECIST 1.1 criteria, Liver metastases, DWI, Intra-rater reliability, Kappa statistic, Cohen’s Kappa, Weighted Kappa

## Abstract

**Background:**

In research designs that rely on observational ratings provided by two raters, assessing inter-rater reliability (IRR) is a frequently required task. However, some studies fall short in properly utilizing statistical procedures, omitting essential information necessary for interpreting their findings, or inadequately addressing the impact of IRR on subsequent analyses’ statistical power for hypothesis testing.

**Methods:**

This article delves into the recent publication by Liu et al. in *BMC Cancer*, analyzing the controversy surrounding the Kappa statistic and methodological issues concerning the assessment of IRR. The primary focus is on the appropriate selection of Kappa statistics, as well as the computation, interpretation, and reporting of two frequently used IRR statistics when there are two raters involved.

**Results:**

The Cohen’s Kappa statistic is typically utilized to assess the level of agreement between two raters when there are two categories or for unordered categorical variables with three or more categories. On the other hand, when it comes to evaluating the degree of agreement between two raters for ordered categorical variables comprising three or more categories, the weighted Kappa is a widely used measure.

**Conclusion:**

Despite not substantially affecting the findings of Liu et al.?s study, the statistical dispute underscores the significance of employing suitable statistical methods. Rigorous and accurate statistical results are crucial for producing trustworthy research.

Assessing inter-rater reliability (IRR) is a common requirement in many research designs, particularly for demonstrating consistency among observational ratings provided by two raters [1]. Unfortunately, some studies misuse statistical procedures, fail to report critical information necessary to interpret their results, or do not adequately address how IRR affects the power of subsequent analyses for hypothesis testing [2]. This matters arising paper examines the recent publication by Liu et al. in *BMC Cancer* [3], highlighting controversy of the Kappa statistic and methodological concerns related to IRR assessment. The focus is on the selection of appropriate Kappa statistics, as well as computation, interpretation, and reporting of two commonly-used IRR statistics between two raters.

## Kappa statistic

Typically, classical statistical techniques like the Kappa statistic, which encompasses Cohen’s Kappa and its adaptations, are utilized to evaluate IRR when dealing with nominal and categorical data.

### Cohen’s kappa

Cohen’s Kappa [4] is a frequently employed classical statistical method to assess IRR, and it’s only suitable for fully-crossed designs with precisely two raters. Moreover, Cohen’s Kappa is commonly used for two raters with two categories or for unordered categorical variables with three or more categories [5, 6]. Ordered variables, also known as ordinal variables, possess a natural ordering or hierarchy among their categories. This means that the categories can be ranked or ordered in a meaningful way based on the magnitude or intensity of the variable being measured. Unordered variables, also known as nominal variables, do not have any inherent ordering or ranking among their categories. A Likert scale is a commonly used measurement tool where respondents rate their agreement or disagreement on a scale, typically ranging from “strongly disagree” to “strongly agree.” In this case, the categories have a clear ordering and represent a continuum of responses. For instance, the scale could be “1. Strongly Disagree, 2. Disagree, 3. Neutral, 4. Agree, 5. Strongly Agree.” Here, the variable is ordered because the categories have a logical sequence and can be ranked based on the level of agreement. On the other hand, let’s consider a variable like eye color, which includes categories such as blue, green, brown, and hazel. Unlike Likert scale ratings, eye color categories do not have a natural order or hierarchy. There is no inherent ranking or meaningful sequence among the categories. Therefore, eye color would be considered an unordered variable.

Cohen’s Kappa is calculated as follows:

1 $${k}_{C}=\frac{\sum _{j=1}^{n}{u}_{jj}\left(i{i}^{{\prime }}\right)-\sum _{j=1}^{n}{p}_{ij}{p}_{{i}^{{\prime }}j}}{1-\sum _{j=1}^{n}{p}_{ij}{p}_{{i}^{{\prime }}j}}$$

The value of ${u}_{jj}\left(i{i}^{{\prime }}\right)$ is the proportion of objects put in the same category *j* by both raters $i$ and ${i}^{{\prime }}$. The value of ${p}_{ij}$ is the proportion of objects that rater $i$ assigned to category $j$.

One limitation of Cohen’s Kappa is its sensitivity to the prevalence of agreement in the data. When the categories being rated are imbalanced or when there is a high prevalence of one category, Cohen’s Kappa tends to be biased and may not accurately reflect the true agreement between raters. Another limitation is that Cohen’s Kappa assumes that the raters are independent, meaning their ratings are not influenced by each other. However, in some cases, raters may be influenced by each other’s ratings, leading to inflated agreement estimates [7]. On the other hand, Cohen’s Kappa has several advantages. It accounts for the agreement that would occur by chance, providing a more accurate measure of agreement between raters compared to simple percent agreement [8]. Cohen’s Kappa also allows for the assessment of agreement beyond chance, considering both the observed agreement and the expected agreement by chance. Additionally, Cohen’s Kappa is applicable to categorical variables with two or more categories, making it a versatile measure for a wide range of research fields. It is important for researchers to be aware of the limitations of Cohen’s Kappa and to consider alternative measures, such as weighted Kappa, when dealing with imbalanced data or when there is potential for rater dependency.

### Weighted kappa

In cases where there is a need to evaluate the level of agreement between two raters regarding ordered categorical variables that consist of three or more categories, the weighted Kappa is frequently utilized as a measure [9]. Weighted Kappa comes in two forms: linear weighted Kappa (LWK) [10] and quadratic weighted Kappa (QWK) [11]. The LWK extends Cohen’s Kappa statistic by attributing different weights to different categories of agreement and disagreement based on the linear distance between the categories on the rating scale [10]. In contrast, the QWK assigns weights based on the quadratic distance between the categories on the rating scale, allowing for a more nuanced analysis of the agreement between raters [11]. Both LWK and QWK are valuable measures of interrater reliability (IRR) as they provide more information about the agreement between raters than Cohen’s Kappa. The choice between the two depends on the specific situation and the data being analyzed. Reporting both LWK and QWK coefficients is recommended in situations where not all disagreements carry equal weight as this can provide a more comprehensive understanding of the distribution of disagreements [12]. Doing so can ensure a more accurate and detailed evaluation of the consistency and reliability of the data, which is particularly crucial when dealing with complex datasets [13].

Weighted Kappa is calculated as follows:

2 $${w}_{ij}^{\left(m\right)}=1-{\left(\left.\frac{\left|i-j\right|}{n-1}\right)\right.}^{m}$$

3 $${k}_{m}=1-\frac{1-\sum _{i=1}^{n}\sum _{j=1}^{n}{w}_{ij}^{\left(m\right)}{p}_{ij}}{1-\sum _{i=1}^{n}\sum _{j=1}^{n}{w}_{ij}^{\left(m\right)}{p}_{i}{q}_{j}}$$

Where m ≥ 1, $p$ and $q$ are relative frequencies, which reflect the proportion of frequency to the number of samples. ${p}_{i}={\sum }_{j=1}^{n}{p}_{ij} and {q}_{i}={\sum }_{j=1}^{n}{p}_{ji}$. In special cases, ${k}_{1}$ is the LWK and ${k}_{2}$ is the QWK.

A limitation of weighted Kappa is its complexity and potential subjectivity in assigning weights [14]. The choice of weights relies on expert judgment or empirical evidence, and different weightings can lead to varying results. Additionally, weighted Kappa requires a clear understanding of the underlying data and the appropriate selection of weighting schemes, which can be challenging. However, weighted Kappa offers several advantages [15]. Firstly, it allows for a more nuanced analysis of agreement, taking into account the severity or importance of disagreements. This is particularly valuable when the categories have different levels of relevance or when certain disagreements are more critical than others. Secondly, weighted Kappa can be useful when dealing with ordinal or interval categorical variables, as it captures the inherent ordering of categories. It provides a more accurate representation of the agreement by considering the magnitude of disagreement. Overall, the advantages of using weighted Kappa lie in its ability to capture the relative importance of disagreements and provide a more comprehensive assessment of agreement. However, it requires careful consideration and application of appropriate weighting schemes, making it essential to interpret the results in conjunction with the specific context and research objectives.

## The statistical controversy over Liu et al.’s article

Liver metastases occur in about 5% of newly diagnosed cancer patients, leading to reduced survival rates. Treatment options include systemic chemotherapy, ablation, and surgery depending on the stage and source of metastasis. Radiological assessment using computed tomography (CT) or magnetic resonance imaging (MRI) is critical in making treatment decisions, with MRI being superior for hepatic metastasis evaluation and diffusion-weighted imaging (DWI) being useful for tumor assessment. The Response Evaluation Criteria in Solid Tumor 1.1 (RECIST 1.1) is the standard method for evaluating tumor response, but it has variability and challenges. To address this, researchers developed computer-aided systems for automated lesion segmentation. Liu et al. [3] proposed a deep learning-based liver metastases segmentation method that assessed treatment response based on RECIST 1.1 and compared the accuracy of automated segmentation to radiologists’ readings. While the authors’ statement had some merit, the approach requires further evaluation.

After reevaluating the Kappa values in the authors’ data, statistical discrepancies were identified in three groups: R1 vs. reference standard in the testing dataset and validation cohort, as well as R2 vs. reference standard in the testing dataset (Table 1). The authors overestimated the agreement between R1 and the reference standard in the testing dataset. The reassessment showed that R1 and the reference standard had fair agreement in the testing dataset. Our analysis indicated fair agreement with a LWK of 0.38 and a QWK of 0.40, which differed from the authors’ reported moderate agreement with a Kappa value of 0.48. Furthermore, our analysis indicated substantial agreement with LWK of 0.67 and QWK of 0.75, differing from the authors’ reported Kappa value of 0.63. On the other hand, the IRR between R2 and the reference standard showed no agreement for *p* > 0.05, contradicting the authors’ report fair agreement with Kappa value of 0.30. We suggest that the authors provide further clarification. Our linear weighted Kappa values were consistent with the other three groups.

**Table 1:** The confusion matrix of the response assessment results with respect to reference standard and the IRRs of treatment response assessment

			Reference standard							
			PR	SD	PD	Total	κ_c_	κ_lW_	κ_qW_	κ*
Testing dataset	R1	PR	1	0	2	3	0.35 (*p* < 0.05, 95% CI = 0.11–0.60)	0.38 (*p* < 0.05, 95% CI = 0.11–0.66)	0.40 (*p* < 0.05, 95% CI = 0.08–0.73)	0.48
		SD	4	3	3	10				
		PD	1	1	16	18				
		Total	6	4	21	31				
	R2	PR	1	0	3	4	0.22 (*p* > 0.05, 95% CI =−0.03–0.47)	0.20 (*p* > 0.05, 95% CI =−0.08–0.48)	0.18 (*p* > 0.05, 95% CI =−0.17–0.53)	0.30
		SD	3	3	5	11				
		PD	2	1	13	16				
		Total	6	4	21	31				
	Automated segmentation	PR	2	0	2	4	0.49 (*p* < 0.05, 95% CI = 0.23–0.76)	0.51 (*p* < 0.05, 95% CI = 0.23–0.79)	0.52 (*p* < 0.05, 95% CI = 0.19–0.85)	0.51
		SD	3	3	1	7				
		PD	1	1	18	20				
		Total	6	4	21	31				
Validation cohort	R1	PR	3	2	0	5	0.57 (*p* < 0.05, 95% CI = 0.34–0.80)	0.67 (*p* < 0.05, 95% CI = 0.46–0.87)	0.75 (*p* < 0.05, 95% CI = 0.54–0.95)	0.63
		SD	3	2	1	6				
		PD	1	0	19	20				
		Total	7	4	20	31				
	R2	PR	2	2	2	6	0.38 (*p* < 0.05, 95% CI = 0.14–0.61)	0.45 (*p* < 0.05, 95% CI = 0.20–0.69)	0.51 (*p* < 0.05, 95% CI = 0.23–0.79)	0.45
		SD	4	2	2	8				
		PD	1	0	16	17				
		Total	7	4	20	31				
	Automated segmentation	PR	4	2	0	6	0.52 (*p* < 0.05, 95% CI = 0.26–0.78)	0.60 (*p* < 0.05, 95% CI = 0.35–0.85)	0.66 (*p* < 0.05, 95% CI = 0.39–0.93)	0.60
		SD	1	2	3	6				
		PD	2	0	17	19				
		Total	7	4	20	31				

The data has been cited from the article published by Liu et al. [3]. R1: an attending radiologist with 8 year’s reading experience; R2: a fellow radiologist with 4 year’s reading experience; κ_c_: Cohen’s Kappa value; κ_lw_: linear weighted Kappa value; κ_qw_: quadratic weighted Kappa value; κ*: Kappa value calculated by Liu et al.; CI: confidence interval; PR: partial response; SD: stable disease; PD: progressive disease

## Conclusion

In summary, Cohen’s Kappa is appropriate for assessing agreement between two raters with two categories or for categorical variables with two categories. Weighted Kappa, specifically LWK or QWK, is employed when dealing with ordered categorical variables with three or more categories, considering the magnitude of agreement and disagreement. The selection between Cohen’s Kappa and weighted Kappa depends on whether the data is categorical or ordered, and whether the research question requires a nuanced analysis of the agreement and disagreement.

Liu et al. [3] assessed the level of agreement between two raters for a set of ordered categorical variables comprising three categories: PR, SD, and PD. Weighted Kappa is a more appropriate option in this scenario as opposed to Cohen’s Kappa. After reevaluating the Kappa values, discrepancies were found in three groups: R1 vs. reference standard in the testing dataset and validation cohort, and R2 vs. reference standard in the testing dataset. The authors underestimated agreement between R1 and the reference standard in the testing dataset, while our analysis showed fair agreement. Our analysis also indicated substantial agreement for R1 with LWK of 0.67 and QWK of 0.75, differing from the authors’ reported Kappa value of 0.63. However, there was no agreement between R2 and the reference standard, contradicting the authors’ report of fair agreement with a Kappa value of 0.30. Although the statistical controversy in Liu et al.’s study does not significantly impact the conclusions of their paper, it emphasizes the significance of addressing and resolving these misunderstandings.

## Data Availability

Not applicable.

## Abbreviations

IRR: Inter-rater reliability
CT: Computed tomography
MRI: Magnetic resonance imaging
DWI: Diffusion-weighted imaging
RECIST: Response Evaluation Criteria in Solid Tumor
LWK: Linear weighted Kappa
QWK: Quadratic weighted Kappa
R1: An attending radiologist with 8 year’s reading experience
R2: A fellow radiologist with 4 year’s reading experience
κ_c_: Cohen’s Kappa value
κ_lw_: Linear weighted Kappa Value
κ_qw_: Quadratic weighted Kappa value
κ*: Kappa value calculated by Liu et al
CI: Confidence interval
PR: Partial response
SD: Stable disease
PD: Progressive disease
